# A Functional Assay for the Determination of Heparin-Induced Thrombocytopenia via Flow Cytometry

**DOI:** 10.3390/diagnostics13183019

**Published:** 2023-09-21

**Authors:** Ingrid Skornova, Tomas Simurda, Lucia Stanciakova, Viliam Lauko, Pavol Holly, Matej Samos, Tomas Bolek, Martin Schnierer, Miroslava Drotarova, Kristina Maria Belakova, Juraj Sokol, Jan Stasko, Marian Mokan, Jaroslav Gumulec, Leona Chrastinova

**Affiliations:** 1 National Center of Hemostasis and Thrombosis, Department of Hematology and Transfusiology, Comenius University in Bratislava, Jessenius Faculty of Medicine in Martin and University Hospital in Martin, 03601 Martin, Slovakia; inkaskornova@gmail.com (I.S.); palhol@gmail.com (P.H.); miroslava.sterankovall@gmail.com (M.D.); belakova31@uniba.sk (K.M.B.); juraj.sokol@uniba.sk (J.S.); jan.stasko@uniba.sk (J.S.); 2 National Institute of Cardiovascular Diseases, 83348 Bratislava, Slovakia; 3 Department of Internal Medicine I, Jessenius Faculty of Medicine in Martin, Comenius University in Bratislava, 03601 Martin, Slovakia; matej.samos@gmail.com (M.S.); ato.bolek@gmail.com (T.B.); mokanmarian@gmail.com (M.M.); 4 Department of Gastroenterology Medicine, Jessenius Faculty of Medicine in Martin, Comenius University in Bratislava, 03601 Martin, Slovakia; martinschnierer@gmail.com; 5 Clinic of Hemato-Oncology, Faculty Hospital in Ostrava, 708 00 Ostrava, Czech Republic; jaromir.gumulec@fno.cz; 6 Institute of Hematology and Blood Transfusion in Prague, 128 20 Nove Mesto, Czech Republic; leona.chrastinova@uhkt.cz

**Keywords:** heparin-induced thrombocytopenia (HIT), heparin–PF4 complex (platelet factor 4), immunological and functional assays, serotonin release assay (SRA)

## Abstract

Heparin-induced thrombocytopenia (HIT) is a life-threatening complication of heparin therapy (both unfractionated heparin and low-molecular-weight heparin). In our study, we examined a group of 122 patients with suspected HIT. The samples of all patients were analyzed in the first step using an immunoassay (ID-PaGIA Heparin/PF4, Hemos1L-Acustar HIT IgG, ZYMUTEST HIA Monostrip IgG) to detect the presence of antibodies against heparin–PF4 complexes (platelet factor 4). When the immunoassay was positive, the sample was subsequently analyzed for HIT with a functional flow cytometry assay, the HITAlert kit, the purpose of which was to demonstrate the ability of the antibodies present to activate platelets. A diagnosis of HIT can be made only after a positive functional test result. In this article, we present an overview of our practical experience with the use of the new functional method of analysis, HIT, with flow cytometry. In this work, we compared the mutual sensitivity of two functional tests, SRA and the flow cytometry HITAlert kit, in patients perceived as being at risk for HIT. This work aims to delineate the principle, procedure, advantages, pitfalls, and possibilities of the application of the functional test HITAlert using flow cytometry.

## 1. Introduction

Heparin-induced thrombocytopenia (HIT) is a dangerous, immunologically mediated adverse drug reaction to heparin [1]. The diagnosis of HIT is based on a combination of clinical findings, including 4Ts scores and laboratory tests. To determine the diagnosis, an evaluation of clinical findings and a laboratory determination of the presence of antibodies causing HIT are necessary [2].

**Figure 1:**
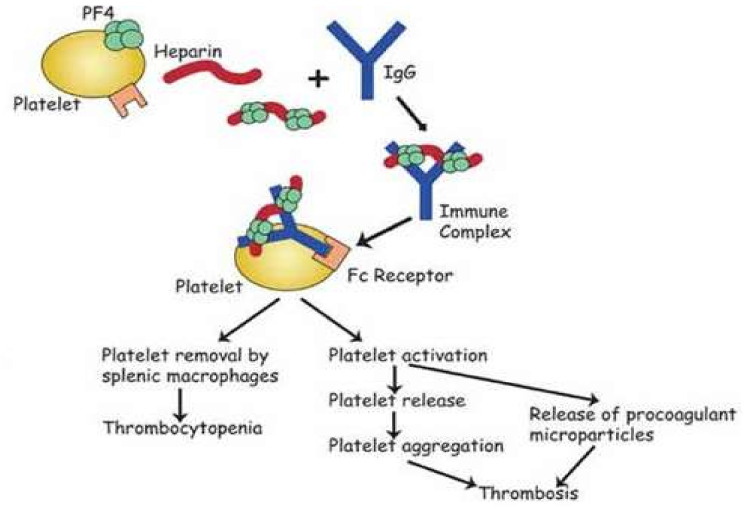
Heparin-induced thrombocytopenia (HIT) caused by autoantibodies against platelet factor 4 (PF4) bound to negatively charged heparin. The heparin–PF4–antibody complex binds to platelets via FcγRII and causes their activation and aggregation; microparticles are released [4].

We distinguish two types of HIT. Type I is a non-immune disorder that is the result of the direct effect of heparin in terms of activating platelets [3,4]. It occurs during the first exposure to heparin, while the number of blood platelets normalizes with continued heparin treatment. HIT type 2 is an immune-mediated disorder that usually occurs 4 to 10 days after heparin exposure. The prevalence is approximately 0.5–5% of heparinized patients [5]. HIT is caused by platelet-activating antibodies of the IgG class that recognize complexes of platelet factor 4 (PF4) and heparin. The heparin–PF4–antibody complex stimulates the platelet receptor FcγRII, which causes platelet activation and aggregation; microparticles are then released. Consumption of platelets in (micro)thrombi as well as liver clearance most probably play an important role. Clinically, thrombocytopenia in HIT is a hypercoagulable state with an increased risk of thrombosis (Figure 1).

Laboratory diagnosis of HIT usually takes place in two steps. The first step is to demonstrate the presence of antibodies against the heparin–PF4 complex using an immunological test; subsequently, in the case of a positive test (i.e., evidence of the presence of antibodies), confirmation or exclusion of HIT is made using a functional test. In a functional test, the antibody’s ability to activate platelets is verified. A definitive diagnosis of HIT can only be established with a positive functional test result, while there is a considerable group of patients (especially after cardiac surgery performance) who have antibodies present (positivity in immunological tests) but have a negative functional test, and therefore they do not have HIT. Using the 4Ts score, a clinical probability scoring system, we can determine the probability of HIT: high probability (6–8 points); intermediate probability (4–5 points); and low probability (3 points) [6]. The 4Ts score and diagnostic and initial treatment algorithms may be used as a guide for clinical diagnosis. In a meta-analysis, the negative predictive value of a low-probability 4T score was 99.8% (i.e., a low-probability score reliably excludes HIT). The positive predictive values of intermediate and high-probability scores were 14% and 64%, respectively [7,8].

The guidelines of the American Society of Hematology recommend the use of the 4Ts score for estimating the pretest probability of HIT; furthermore, it is also recommended to avoid HIT laboratory testing and empirical treatment in patients with a low-probability 4Ts score [9].

## 2. Materials and Methods

The design of this study is a retrospective cohort study. This study was conducted from April 2017 to September 2022, and we analyzed 122 samples from patients with suspected HIT. The samples were obtained from several centers. Thirty samples (Table 1) were obtained at the National Institute of Cardiovascular Diseases in Bratislava. The other 92 samples were obtained from the University Hospital in Martin, Slovakia.

The tests were performed in multiple labs. For a batch of 30 samples (Table 1), we determined each sample’s 4Ts score and results from two immunological assays (the ID-PAGIA immunoassay and the HemosIL AcuStar HIT-IgG) at the National Institute of Cardiovascular Diseases in Bratislava, and SRA was performed in a laboratory at the Institute of Hematology and Blood Transfusion in Prague. The ELISA assay was performed in the laboratory of the Clinic of Hematooncology, Faculty Hospital in Ostrava, Czech Republic.

### 2.1. Samples

#### 2.1.1. Donor Samples

Blood samples from O-blood-type healthy donors were obtained in sodium citrate tubes (BD Vacutainer, BD-Plymouth, Pl67BP.UK). We processed donor venous blood directly after drawing. The blood was centrifuged for 5 min at 100× *g* with low acceleration and the brake off. The platelet-rich plasma (PRP) was used within 2 h. It was important that the platelet donor did not use platelet inhibitors, like aspirin, or anti-inflammatory drugs, like ibuprofen, Advil, etc., during the last 10 to 14 days prior to the blood draw.

#### 2.1.2. Patient Samples

The patient samples were collected in two test tubes (one tube containing sodium citrate and the second without). The sample without an anticoagulant agent was left for 30 min to allow clot formation, after which we spun the tube for 20 min at 1000 g at room temperature (RT). The serum sample was optimally processed within 12 h after collection. The serum was used for the immunoassays ID-PaGIA, HIT *Alert* assay, and SRA. The plasma samples were prepared from blood collected in tubes containing sodium citrate as an anticoagulant and were directly analyzed or stored at –20 °C. The collected plasma was used for the immunoassays ZYMUTEST HIA Monostrip IgG and HemosIL Acustar IgG.

### 2.2. Immunological Assays

#### 2.2.1. Particle Gel Immunoassay

The ID-PAGIA immunoassay (Bio-RAD, DiaMed GmbH, Cressier, Switzerland) uses red, high-density polystyrene beads to bind heparin–PF4 complexes. We left the serum samples for 5 min to react with the heparin–PF4 complexes, then we centrifuged for 10 min at 910× *g* and visually read the reaction results.

Non-agglutinated beads indicated the absence of antibodies, while in the presence of anti-heparin–PF4 antibodies, agglutinated beads were observed on the gel’s surface.

#### 2.2.2. Chemiluminescence Assay

The HemosIL AcuStar HIT-IgG_PF4-H (Instrumentation Laboratory, Bedford, MA, USA) assay is a chemiluminescent immunoassay comprising magnetic particles coated with PF4–polyvinyl sulfonate (PVS) complexes that capture heparin–PF4 antibodies in the sample. We followed as per the manufacturer’s instructions; we added a sample tracer comprising an isoluminol-labeled anti-human IgG antibody and reagents that trigger the luminescent reaction; the emitted light was measured as relative light units (RLUs) using the ACL AcuStar optical system. The RLUs were directly proportional to the heparin–PF4 IgG concentration in the sample.

#### 2.2.3. Enzyme-Linked Immunosorbent Assay (ELISA)

ZYMUTEST HIA Monostrip IgG (HYPHEN BioMED, Neuville sur Oise, France) is an ELISA immunological assay. The plasma to be tested is diluted with HIA diluent at a ratio of 1:100. First, 50 µL cell lysate and 200 µL tested plasma (diluted 1:100) are pipetted into the well, on the surface of which biologically available unfractionated heparin is bound. If heparin-dependent antibodies are present, they form complexes with the fixed heparin. After a 60-min incubation and washing, 200 μL of the immunoconjugate is pipetted into the wells and allowed to incubate for 60 min at a temperature of 18–25 °C. After adding the substrate, tetra-methylbenzidine, and hydrogen peroxide, the reaction forms a blue solution. The reaction is stopped by adding sulfuric acid, causing the color to change to yellow. The intensity of the staining is directly proportional to the concentration of heparin-dependent antibodies present in the sample. Spectrophotometric detection of the color intensity was carried out at 450 nm. During the entire procedure, the temperature range was maintained within 18–25 °C.

### 2.3. Functional Assays

#### 2.3.1. Serotonin Release Assay (SRA) using HPLC and Fluorometric Detection

Platelets from healthy donors were washed using Tyrode buffer without calcium ions and resuspended in Tyrode buffer (pH 7.4) to a final concentration of 300,000/µL. The serum samples were incubated at 56 °C for 30 min. We incubated 20 µL of patient serum, 75 µL of washed platelets, and 5 µL of heparin/fraxiparine solution (0.1 IU/mL and 100 IU/mL final concentrations, respectively) under gentle mixing for 60 min at RT. The reaction was terminated by adding EDTA (13.4 mM). The supernatant of the reaction mixture was treated using sodium borohydride (2.64 M) and perchloric acid (3.84 M), centrifuged, and 20 µL of supernatant was injected into a NUCLEOSIL C18 column (125 × 3 mm, 5 μm). The mobile phase consisted of methanol, 100 mM H3PO4, and 5 mM hexanesulfonic acid sodium salt (10:90, *v*/*v*) and was adjusted to pH 2.5 with triethylamine. The chromatography was performed isocratically with a flow rate of 0.5 mL/min at 45 °C using fluorimetric detection at excitation 270 nm and emission 340 nm. The percentage of released serotonin was calculated using the total serotonin concentration in donor platelets. A patient serum was considered positive for HIT antibodies if the difference in % released between the suspension with 0.1 IU/mL and 100 IU/mL heparin or fraxiparine, respectively, was higher than 20%.

#### 2.3.2. Flow Cytometry Platelet Activation Assay

For the HITAlert assay, donor platelets (PRP) are used, which are incubated with patient serum in the presence or absence of heparin. When pathogenic antibodies are present, the activation of the donor platelets is shown using a platelet activation marker.

We used a DxFLEX flow cytometer (PN C44966AA, Beckman Coulter, Nyon, Switzerland) to measure platelet activation [10].

First, we made sure that the flow cytometer was calibrated correctly according to the manufacturer’s instructions. For the adjustment of the flow cytometer settings, we used three tubes: We added 5 µL of the assay sample—donor platelets and staining buffer—to each tube. Then a platelet marker was added (monoclonal antibody) to the second tube and a platelet activation marker (recombinant protein) to the third tube. We mixed the tubes and then incubated them for 15 min in the dark at RT. We generated three dot plots: a forward scatter (FSC) vs. side scatter (SSC) dot plot with a logarithmic scale to select the platelets; a R-PE vs. SSC dot plot to select the platelet marker positive events; and a FITC vs. R-PE dot plot to determine the activation of the platelets. We adjusted the voltage settings for the FSC–SSC using a tube containing the sample and buffer only. A second and third tube were used to adjust the compensation. These compensation settings between the FITC (FL-1) and R-PE (FL-2) fluorescence signals were optimized to effectively separate the stimulated (FL-1 positive) and unstimulated (FL-1 negative) platelets. We collected list mode files of at least 10,000 events for FSC, SSC, and fluorescence signals for both fluorochrome-conjugated antibodies within the gated region of platelets (SSC/R-PE). Fewer than 10,000 events would begin to influence the accuracy of the assay.

When analyzing the HITAlert kit functional test via flow cytometry (IQ Products, Rozenburglaan, Groningen, The Netherlands), we evaluated five test tubes with the following contents:

Test tube I: A sample of donor PRP with heparin, which should show any background activation due to handling unless the PRP donor is HIT-positive. A sample of unstimulated donor platelets should have no more than 1% activation-marker-positive platelets. If this percentage is higher than 5%, the test should be repeated, preferably with platelets from another donor.

Test tube II: A sample of donor PRP with calcium ionophores, which should contain activated thrombocytes that can be used to set the flow cytometer. The stimulated sample should have more than 80% activated platelets. A percentage lower than 80% may be due to the incomplete dissolution of the calcium ionophores. Test tube III: A sample of donor PRP with patient serum, which should show “background“ activation due to the serum. Test tube IV: A sample of donor PRP with patient serum and a physiological concentration of heparin, which should show activation due to the presence of heparin-complex-binding antibodies. Test tube V: A sample of donor PRP with patient serum and an excess of heparin, which should show a decrease in platelet activation in the case of a positive result for test tube IV since immune complexes are disrupted due to the high concentration of heparin.

The results of the patient blood sample evaluation are a qualitative and reliable source to determine the presence of heparin-complex-specific pathogenic antibodies in peripheral blood.

**Figure 2:**
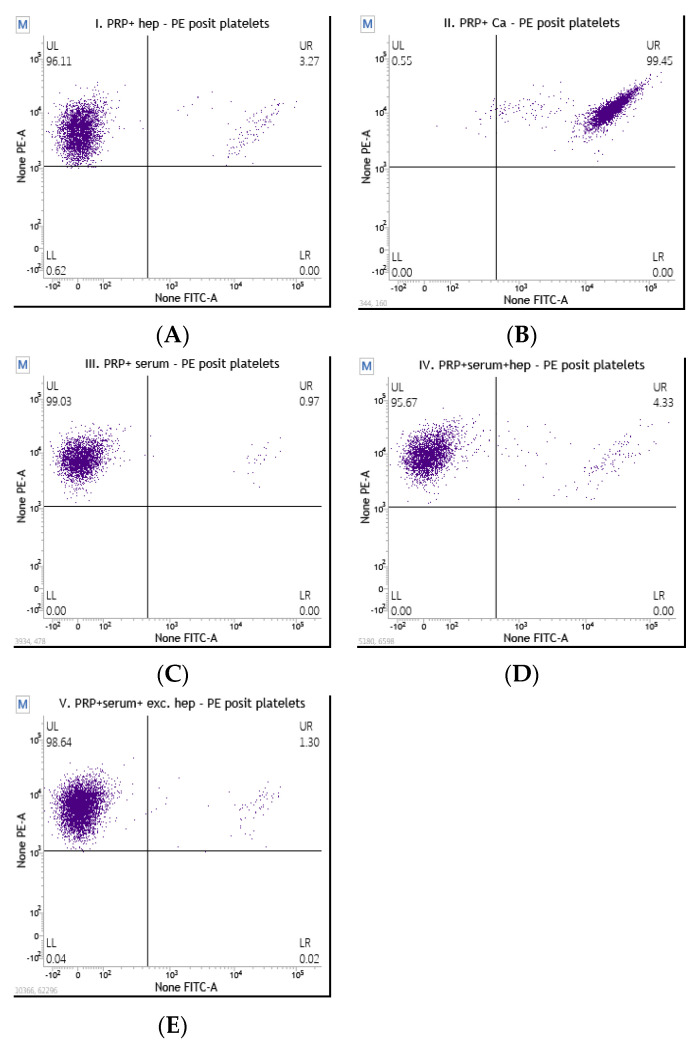
(**A**–**E**) Analysis of a sample from a patient with suspected HIT and a negative result. (**A**): Dot plot I: unstimulated donor platelets; 3.2% of platelets are activated. (**B**): Dot plot II: Ca-ionophore-stimulated donor platelets; 99% activated. (**C**): Dot plot III: donor platelets in patient serum without heparin; 0.97% activated. (**D**): Dot plot IV: donor platelets, patient serum, and heparin; 4% activated. (**E**): Dot plot V: donor platelets and patient sample with excess heparin; 1.30% activated.

An analysis of samples from a patient suspected of having HIT with a negative result is shown in Figure 2. On dot plot I without stimulation, we can see that 3.2% of donor platelets were activated; on dot plot II, after their stimulation with Ca ionophores, 99% of platelets were activated. The donor platelets met the required criteria, and we used them to analyze the samples via the HITAlert functional test using flow cytometry. On dot plot III, we have a mixture of donor platelets and patient serum, which resulted in 0.97% activation and thus meets the requirement for HIT-negative patients. Dot plot IV represents the analysis of donor platelets, patient serum, and heparin, resulting in 4% activated platelets. Similarly, dot plot V shows the results of a patient negative for HIT.

**Figure 3:**
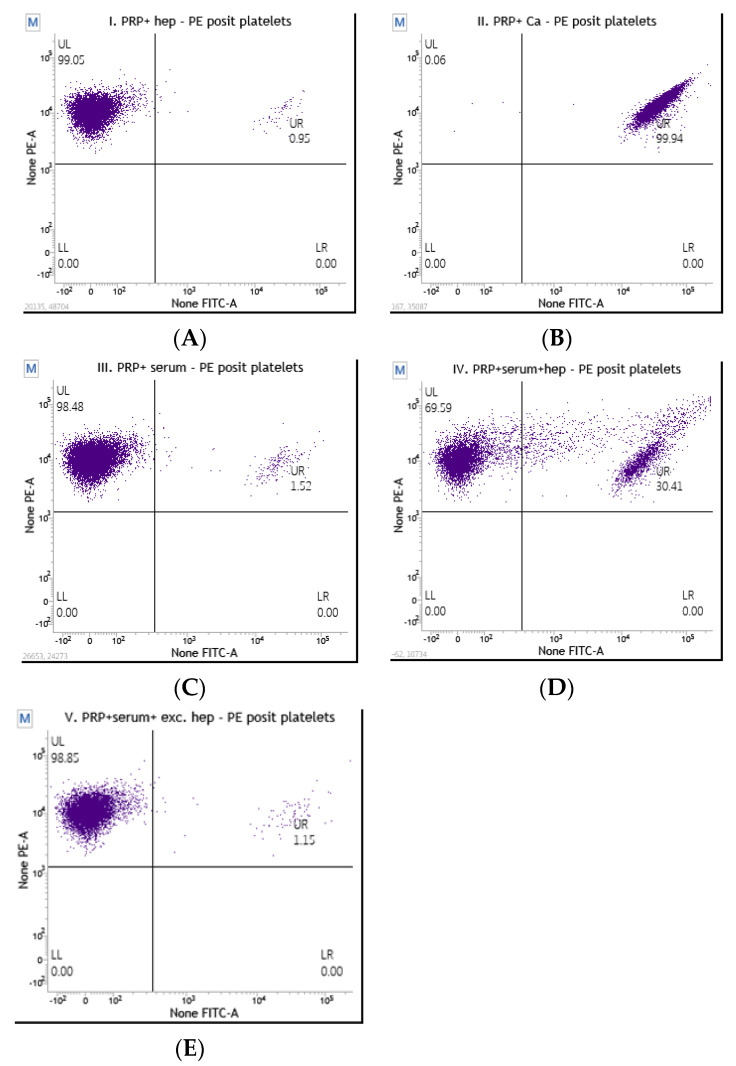
(**A**–**E**) Analysis of a sample from a patient with suspected HIT and a positive result. (**A**): Dot plot I: unstimulated donor platelets; 0.95% of platelets are activated. (**B**): Dot plot II: Ca-ionophore-stimulated donor platelets; 99% activated. (**C**): Dot plot III: donor platelets in patient serum without heparin; 1.52% activated. (**D**): Dot plot IV: donor platelets, patient serum, and heparin; 30.4% activated. (**E**): Dot plot V: donor platelets, patient serum, and excess of heparin; 1.15% activated.

An analysis of samples from a patient suspected of having HIT with a positive result is shown in Figure 3. In this case, we also confirmed that we could use the donor platelets for further analysis with the HITAlert functional test. The donor platelets themselves were not activated but were sufficiently activated after stimulation. However, we can see that in the test tube containing donor platelets, patient serum, and heparin, there was an activation of 30.4%. This means that the patient is very likely to be positive for HIT, while sample III shows an activation less than half that of sample IV.

According to interpretation, the first patient is very likely to be HIT-positive and should have a platelet activation >8% in test tube IV (including donor PRP, heparin, and patient serum), while a HIT-negative patient should have a platelet activation <5% in test tube IV. If the patient has transient activation (5–8%) in tube IV, it is necessary to repeat the analysis with another donor sample [11,12,13,14,15,16,17].

### 2.4. Statistical Analysis

Based on the input contingency table, sensitivity, specificity, positive predictive value, and negative predictive value were calculated using the yardstick library [18] in the statistical software R ver. 4.0.5 [19].

## 3. Results

### 3.1. Patient Characteristics

#### 3.1.1. Enrolled Patients

A total of 122 patients with a median age of 70 (21–98) were enrolled in our study. Of these, there were 52 females and 70 males (Table 1).

All enrolled patients were hospitalized and, in most cases, had a normal platelet count above 140 × 10^9^/L. Only 6 patients out of a total of 122 used heparin before hospitalization. During continued treatment with heparin, the number of thrombocytes decreased in all of them. All 122 patient samples were analyzed with the ID-PaGIA Heparin/PF4 immunoassay, on the basis of which 98 (80%) were positive, 8 (7%) were negative, and 16 (13%) were weakly positive results. At the same time, all 122 samples were tested via flow cytometry using the HITAlert kit, with 39 (32%) positive results and 83 (68%) negative results. The results for all patients are shown in Table 1.

#### 3.1.2. The Selected Number of Patients with a Detailed Clinical Description

Our group of 122 patients included 30 patients from the National Institute of Cardiovascular Diseases in Bratislava. These included 11 females with a median age of 73 (54–85) and 19 males with a median age of 64 (21–92) with a detailed clinical description. Five patients (20%) overcame thrombosis. The patients had a normal platelet count. With the ongoing treatment with heparin, the patients’ thrombocytes decreased below 140 × 10^9^/L. Thrombocytopenia was one of the criteria for determining the 4Ts score. Prospective data for the calculation of the 4Ts score were collected in the form of a standardized questionnaire filled out by the attending physician, while the subsequent calculation of the 4Ts was performed by a hematologist based on the data from the questionnaire.

Clinical characteristics of these 30 patients according to the underlying diagnosis: In our study, 17 patients were undergoing extracorporeal circulation due to cardiac surgery using unfractionated heparin (UFH) and subsequent treatment with heparin; 4 patients were treated with heparin for deep venous thrombosis; 2 patients were post-angiosurgery with peri-operative administration of heparin and subsequent treatment with heparin; 1 patient underwent transfemoral aortic valve replacement (TAVI) with periprocedural heparin administration and subsequent heparin treatment; 1 patient underwent transapical aortic valve replacement (i.e., cardiac surgery without extracorporeal circulation, but with peri-operative heparin administration) and subsequent ECMO heparin treatment; 1 patient with arrhythmia was treated with a heparin bypass as a part of the prevention of cardioembolic CMP; 1 patient was post-percutaneous coronary intervention (PCI) with coronary stent implantation and peri-procedural heparin administration as well as subsequent heparin treatment; and 2 patients underwent diagnostic angiography with peri-procedural administration of heparin, most likely in the subacute phase of previously diagnosed and now overlooked HIT before angiography.

Type of treatment: Eleven patients were treated with UFH, three with low-molecular-weight heparin (LMWH), and sixteen with UFH and LMWH (not at the same time). All 30 patients in this group were tested with three immunological tests (ZYMUTEST HIA Monostrip IgG, HemosIL AcuStar HIT-IgG_PF4-H, and gel immunoassay ID-PaGIA) and two functional tests (SRA and HITAlert) with the following results: Immunological tests: chemiluminescence (Hemos1L-Acustar HIT IgG): 27 positive, 3 negative; ELISA (ZYMUTEST HIA Monostrip IgG): 25 positive and 5 not tested; gel immunoassay (ID-PaGIA): 30 positive. Functional tests: serotonin release assay (SRA): 23 positive, 2 negative, and 5 unexamined; HITAlert: 19 positive and 11 negative. The results are shown in Table 1. A summary table of the HIT antibody testing results is shown in Table 2.

An SRA test was also performed on this group of patients, the results of which were compared with the HITAlert test. We determined the sensitivity of the HITAlert test to be 70%.

## 4. Discussion

Rapid diagnostic evaluation of suspected heparin-induced thrombocytopenia (HIT) is critical to guide initial patient management. We analyzed all patients in our study with the ID-PaGIA-H/PF4 immunological test. This is a rapid antibody test that provides fast and reliable results concerning the exclusion of HIT. This test has excellent negative predictive value and gives results in less than 15 min. We also analyzed 30 patients from the monitored group with two other immunoassays.

While there was generally good agreement between the different immunological methods, several discordances were noted. For example, we observed one false-positive HIT case using ID-PaGIA and ELISA and one false-positive HIT case using all three immunoassays (Table 1), using the SRA as the diagnostic gold standard. We identified two probable false negatives for HIT using the chemiluminescence method.

Immunological assays should be used to screen for HIT ahead of functional assays for all intermediate and high-probability HIT patients as well as the occasional low-probability patient according to their 4Ts scores where clinical suspicion remains sufficiently high. Which immunological assay a laboratory will select for HIT screening will depend on locally available/approved technologies [20]. However, in practice, it is not always possible to determine the 4Ts score due to missing data, and not all doctors determine it.

The obtained data from the group of patients (Table 1) support the work of Solana et al. [21] in determining HIT in clinical practice using the 4Ts score to ensure testing and clinical management. Similar to the study by Solana et al. [21], all our patients with a high risk according to the 4Ts scores were positive according to the SRA methods.

Similar conclusions were described by Favaloro [22], stating that a low 4Ts score usually excludes HIT but that this may not always be true; for example, one patient in our study with a 4Ts score of 2 was SRA-positive. Favaloro also states that even a high 4Ts score does not always prove HIT. The 4Ts scoring system is a simple and commonly used tool for assessing the risk of HIT [23,24]. In our patient group (Table 1), all patients with high and intermediate 4Ts scores had a positive SRA assay, with only one patient having a negative SRA assay. In the literature, the SRA is considered the gold standard, and so we compared the results of the HITAlert functional test with the results of the SRA. By comparing the SRA results, we identified false-negative HIT cases with the HITAlert kit in seven cases. Despite the fact that our group only had 25 patients in whom we could compare both functional tests, we can state that utilizing HITAlert has a significant benefit in diagnosis.

Table 1B patients did not have a calculated 4Ts score or an examined SRA test, so we could not compare the results of the functional tests in the cases.

## 5. Conclusions

The goal of introducing the HITAlert functional test into practice was to provide a complete diagnosis of HIT. Based on our experience with the functional HIT test using flow cytometry, we can say that the method has its advantages. The analysis time is relatively short; it takes up to 2 h. But even this test cannot be applied in all environments as it requires a flow cytometer and expert knowledge. The value of its sensitivity, which we obtained by comparison with the SRA test, is comparable to the value given in the HITAlert package insert −78%. The disadvantage is the need for donor platelets, which must not be activated; they did not use platelet inhibitors or anti-inflammatory drugs. Based on our experiences mentioned above, we recommend using this method for the functional determination of HIT in laboratories with appropriate instrumentation.

HIT is life-threatening, warranting prompt recognition and intervention. HIT typically presents with thrombosis, more commonly venous thrombosis; however, arterial thrombosis has also been reported. Chittal et al. reported a rare case of arterial and venous thrombosis from delayed-onset HIT [25]. In our group of 30 patients, we diagnosed venous thrombosis in 3 patients and arterial thrombosis in 2, while 19 patients did not have thrombosis and possible thrombosis could not be determined in 6 patients. Hogan and Berger reviewed the pathogenesis, incidence, diagnosis, and management of HIT. Hvas et al. investigated the pathophysiology of HIT treatment using thrombin inhibitors or factor Xa inhibitors [26,27].

In conclusion, determining the presence of HIT-causing antibodies is necessary but not sufficient for the diagnosis of HIT. The available tests have different sensitivities and specificities, and it is important that the laboratory test results are interpreted in the relevant clinical context [12,28,29,30,31,32,33,34]. The analyzed HIT functional test results provide qualitative and reliable data for determining the presence of antibodies specific to the heparin–PF4 complex; however, they are not always 100% precise. The timely and accurate results of immunological and functional tests, together with clinical findings and 4Ts scores, could significantly improve the management and prognosis of this disease.

## Figures and Tables

**Table 1:** Results for patients with suspected HIT. (**A**) The 4Ts scores and clinical evaluation of the patients; and (**B**) without clinical evaluation criteria.

(A)									
No.	Sex	Year of Birth	Thrombosis	4Ts Score	ID-PaGIA	HemosIL Acustar IgG (Cut Off =1 U/mL)	ELISA	SRA	HITAlert
1	M	1979	VT	8	POSITIVE	41.86	POSITIVE	POSITIVE	POSITIVE
2	F	1942	no	7–8	POSITIVE	8.81	POSITIVE	POSITIVE	POSITIVE
3	M	1975	AT	7–8	POSITIVE	21.6	POSITIVE	POSITIVE	POSITIVE
4	M	1949		7–8	POSITIVE	28.57	POSITIVE	POSITIVE	POSITIVE
5	F	1950	VT	7	POSITIVE	1.13	POSITIVE	POSITIVE	NEGATIVE
6	M	1953	no	4–6	POSITIVE	0.76	POSITIVE	POSITIVE	POSITIVE
7	F	1938	no	5	POSITIVE	5.83			POSITIVE
8	F	1954	no	5	POSITIVE	33.97	POSITIVE	POSITIVE	POSITIVE
9	M	1949	no	5	POSITIVE	5.5	POSITIVE	POSITIVE	POSITIVE
10	M	1984	AT	5	POSITIVE	1.94	POSITIVE	POSITIVE	NEGATIVE
11	M	1963	no	5	POSITIVE	2.77	POSITIVE	POSITIVE	NEGATIVE
12	M	1959	no	5	POSITIVE	2.4	POSITIVE	POSITIVE	NEGATIVE
13	M	1973		5	POSITIVE	2.66			NEGATIVE
14	M	1962	VT	4–5	POSITIVE	22.3	POSITIVE	POSITIVE	NEGATIVE
15	M	2002	no	4–5	POSITIVE	0.99	POSITIVE	NEGATIVE	NEGATIVE
16	F	1953	no	4	POSITIVE	53.67	POSITIVE	POSITIVE	POSITIVE
17	M	1931	no	4	POSITIVE	1.9	POSITIVE	POSITIVE	POSITIVE
18	F	1969	no	4	POSITIVE	1.36	POSITIVE	POSITIVE	POSITIVE
19	F	1950	no	4	POSITIVE	25.99	POSITIVE	POSITIVE	POSITIVE
20	F	1941	no	3	POSITIVE	2.66	POSITIVE	POSITIVE	POSITIVE
21	F	1940	no	4	POSITIVE	22.6	POSITIVE	POSITIVE	POSITIVE
22	M	1953	no	4	POSITIVE	16.4	POSITIVE	POSITIVE	POSITIVE
23	M	1957		4	POSITIVE	31.67	POSITIVE	POSITIVE	POSITIVE
24	M	1943	no	3–4	weak positive	16.86	POSITIVE	POSITIVE	POSITIVE
25	M	1964	no	3–4	POSITIVE	2.51	POSITIVE	POSITIVE	NEGATIVE
26	M	1953	no	2	POSITIVE	3.15	POSITIVE	NEGATIVE	NEGATIVE
27	M	1937			POSITIVE	POSITIVE			POSITIVE
28	M	1990			POSITIVE	POSITIVE			POSITIVE
29	F	1942	no		POSITIVE	0.95	POSITIVE	POSITIVE	NEGATIVE
30	F	1954			POSITIVE	POSITIVE			NEGATIVE
**(B)**									
**No.**	**Sex** **M/F**	**Count**	**Year of Birth Median (SD)**			**ID-PaGIA**		**HITAlert**	
31–50	M	8/20	1951 (10.5)			POSITIVE		POSITIVE	
	F	12/20	1951 (11.7)						
52–99	M	29/49	1958 (14.3)			POSITIVE		NEGATIVE	
	F	20/49	1946 (11.0)						
101–114	M	10/15	1952 (18.7)			weak positive		NEGATIVE	
	F	5/15	1953 (14.0)						
116–122	M	4/8	1951 (9.2)			NEGATIVE		NEGATIVE	
	F	4/8	1956 (26.4)						

No.: sample number; AT: arterial thrombosis; VT: venous thrombosis.

**Table 2:** Summary table of the number of individuals with negative, positive, or weakly positive results in the monitored group of patients.

Patient Category	PaGIA	HemosIL Acustar IgG (Cut Off = 1 U/mL)	ELISA	SRA	HITAlert
Total number tested (n)	122	30	25	25	122
Positive	98	27	25	23	39
Negative	8	3	0	2	83
Equivocal (or weak positive)	16	0	0	0	0
Not tested from the total number 122 patients	0	92	97	97	0

## Data Availability

All the data are available from the corresponding author (tomas.simurda@uniba.sk) upon reasonable request.
